# Excessive Sleep and Lack of Sleep Are Associated With Slips and Falls in the Adult Korean Population: A Population-Based Cross-Sectional Study

**DOI:** 10.1097/MD.0000000000002397

**Published:** 2016-01-29

**Authors:** So Young Kim, Sung-Gyun Kim, Songyong Sim, Bumjung Park, Hyo Geun Choi

**Affiliations:** From the Department of Otorhinolaryngology-Head and Neck Surgery and Cancer Research Institute, Seoul National University College of Medicine, Seoul (SYK); Department of Internal Medicine, College of Medicine, Hallym University, Anyang (S-GK); Department of Statistics, Hallym University, Chuncheon (SS); and Department of Otorhinolaryngology-Head and Neck Surgery, Hallym University Sacred Heart Hospital, Anyang, Korea (BP, HGC).

## Abstract

Few studies have evaluated the impacts of excessive sleep duration on falls. This study investigated the associations between sleep duration and falls among Korean adults in a wide range of age groups while adjusting for numerous confounding factors. Data collected from study participants ranging in age from 19 to 109 years old were analyzed from the 2013 Korean Community Health Survey (KCHS). Sleep duration was divided into 5 groups: ≤5, 6, 7, 8, and ≥9 hours per day. The relations between sleep duration and falls (≥1 time or ≥2 times per year) were analyzed using simple and multiple logistic regression analyses with complex sampling. Age, sex, days of vigorous or moderate physical activity, income, education, alcohol use, smoking, stress, obesity, hypertension, diabetes mellitus, hyperlipidemia, stroke, angina or myocardial infarction, arthritis, and asthma were controlled for as confounding factors. Associations between sleep duration and falls were analyzed in 19 to 40, 41 to 60, and 61+ year age groups. Furthermore, the relations between sleep duration and indoor versus outdoor falls were analyzed. Both ≤6 and ≥8 hours of sleep per day were significantly associated with an increased incidence of falls (≥1 time and ≥2 times per year) in the overall adult population (*P* < 0.001 in both instances). In a subgroup analysis, sleep durations of ≤5 and ≥9 hours were significantly associated with an increased incidence of falls (≥1 time a year) in each age group. Six hours of sleep was not significantly associated with falls (≥2 times per year) in the 61+ year age group, and 8 and 9 hours of sleep were not significantly associated with falls (≥2 times a year) in the 19 to 40 year age group. This study demonstrated that long as well as short sleep durations are associated with an increased incidence of falls. However, these relations were not evident in elderly populations with short sleep durations or in young adults with long sleep durations.

## Abstract 2

Supplemental Digital Content is available in the text

## INTRODUCTION

In general, 7 to 8 hours of sleep per night is considered sufficient for adults. Sufficient sleep duration is important for maintaining good health, both physically and psychologically. Insufficient sleep is associated with several adverse health outcomes, including obesity,^1^ hypertension,^2^ diabetes, and metabolic syndrome.^3,4^ In addition, several studies suggest that sleep deprivation is associated with long-term memory loss, traffic accidents, and falls.^5,6^ Our previous research has demonstrated that sleep deprivation is associated with bicycle accidents and fall-related injuries in adolescents.^7^ However, excessive sleep can also lead to poor health outcomes. Many studies have demonstrated a U-shaped relation between sleep duration and chronic diseases. Previous research has shown that long (>8 hours of sleep per night) as well as short (≤6 hours of sleep per night) sleep durations are associated with certain disorders, such as type 2 diabetes^8^ and metabolic syndrome.^9^

Recommended sleep durations vary according to age group. The National Sleep Foundation sleep duration recommendations state that 7 to 9 hours is an appropriate sleep duration for young adults and adults, whereas 7 to 8 hours of sleep is recommended for older adults. These variations in sufficient sleep durations reflect the differences in sleep structure for different age groups. Aging results in a lower quantity and quality of sleep.^10^ As individuals grow older, sleep duration decreases while waking frequency increases.^11–13^ Because of these differences, adverse effects resulting from inadequate sleep duration are also influenced by age. Previous research has reported differences in the relation between sleep duration and metabolic syndrome according to age.^14^ For instance, longer sleep durations (≥9 hours) were associated with metabolic syndrome only in young and middle-aged adults but not in the elderly.^14^ However, to our knowledge, no study has investigated the association between sleep duration and falls stratified by age group.

Additionally, although several studies have examined the relation between sleep deprivation and falls or accidents, little attention has been given to the effects of excessive sleep on falls. Because there are numerous confounding factors associated with falls, it is necessary to adjust for these factors before analyzing the correlation between sleep duration and falls. Thus, the present study aimed to analyze the effect of sleep duration on falls, and the results were adjusted to control for various confounding factors among a large representative population of Koreans. To consider the possible effect of age on study outcomes, we also conducted a subgroup analysis according to age group. To our knowledge, this is the first study on the effect of excessive as well as short sleep durations on falls while considering numerous covariates and a wide range of age groups.

## METHODS

### Study Population and Data Collection

This study was approved by the Institutional Review Board of Korea Centers for Disease Control and Prevention (IRB No. 2013-06EXP-01-3C). Written informed consent was obtained from all the participants prior to conducting the survey.

This study used a cross-sectional study design drawing upon data from the 2013 Korean Community Health Survey (KCHS). Survey data were collected by the Korea Centers for Disease Control and Prevention. The survey gathers information from respondents utilizing face-to-face, paper-assisted personal interviews conducted by trained interviewers. The sample size for the KCHS is 900 subjects in each of the 253 community units, which includes 16 metropolitan cities and provinces. The KCHS uses a 2-stage sampling process. In the 1st stage, a sample area (tong/ban/ri) is selected as the primary sample unit, which is selected according to the number of households in the area using a probability proportional to the sampling method. In the 2nd stage, the number of households in the selected sample tong/ban/ri is identified to create a household directory. Sample households are selected using systematic sampling methods. This process is used to ensure that the sample units are representative of the entire population.^15^ For the sample to be statistically representative of the population, the data collected from the survey are weighted by statisticians based on the sample design.^16^

**FIGURE 1:**
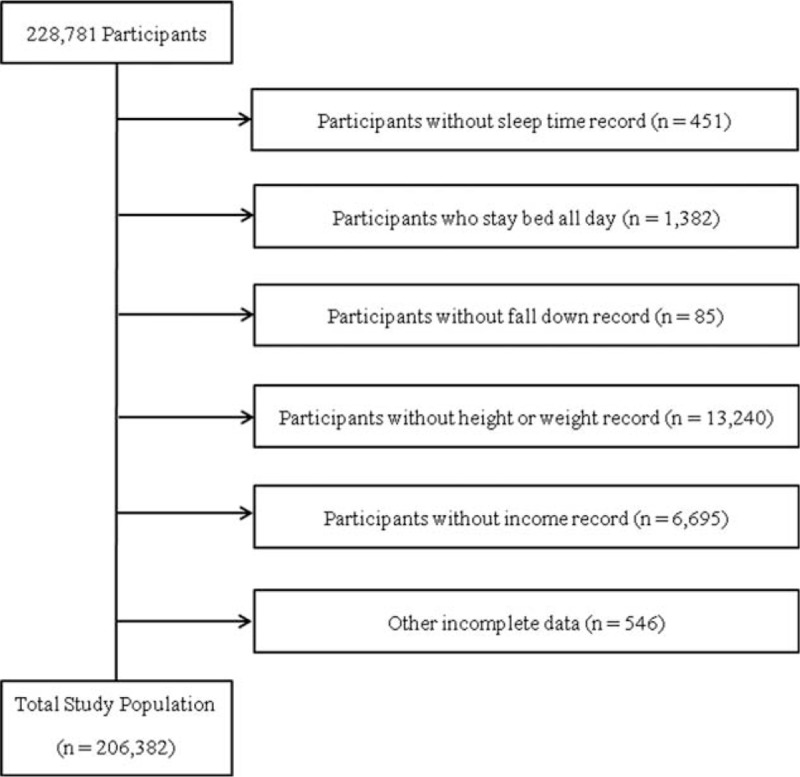
A schematic illustration of participant selection in the present study. Among a total of 228,781 participants, participants who provided records of sleep duration, falls, height or weight, and income were selected for this study. Participants confined to bed all day or who provided incomplete data were excluded. Finally, data for the 206,382 participants from whom complete data were obtained were analyzed.

Of a total of 228,781 participants ranging from 19 to 109 years old, we excluded the following participants in this study: participants who did not respond to sleep duration-related survey items (451 participants); participants who were confined to bed all day (1382 participants); participants who did not report a slip or fall (85 participants); participants who did not respond to weight- or height-related survey items (13,240 participants); participants who did not report income (6695 participants); and participants who had incomplete data regarding exercise history, education level, smoking, alcohol consumption history, stress level, hypertension, diabetes mellitus, hyperlipidemia, stroke, angina or myocardial infarction, arthritis, or asthma history (546 participants). After the exclusion of these participants, a total of 206,382 subjects were included in this study (Figure 1).

### Survey

Sleep duration was divided into 5 groups: ≤5, 6, 7, 8, and ≥9 hours per day. Participants who slept less than 3 hours or more than 12 hours were excluded from the study.

Participants were asked “How many times did you slip or fall in the past year?” Participants who had a history of slips or falls ≥1 time per year were recorded as positive. Because we assumed that anyone can slip or fall by chance without a physiological cause, secondary analysis was performed based on a history of slips or falls ≥2 times per year. For the subgroup analysis, falls were separated according to slip or fall location. Fall locations were divided into 2 groups: indoors (washroom, bedroom, living room, kitchen, floor, or other inside) and outdoors (farming or fishing area, transport area, place of exercise, business district, or other outdoor area). The participants who fell indoors or outdoors ≥1 time per year were recorded as positive.

To measure physical activity, participants were asked about the number of days they had participated in vigorous exercise with considerable shortness of breath for more than 10 minutes in the previous week. Participants were also asked about number of days they had participated in moderate exercise with slight shortness of breath for more than 10 minutes in the previous week. Using methods recommended by the Organization for Economic Cooperation and Development^17^ (ie, dividing household income by the square root of the number of household members), subjects were divided by monthly income into either the lowest, low-middle, upper-middle, or highest quartiles. To explore the influence of educational attainment, uneducated participants and those who had graduated only from elementary or middle schools were assigned to the “low” education group, whereas those who attended high school comprised the “middle” group. Junior college graduates, graduate school, and college graduates formed the “high” education group. Alcohol consumption was divided into the following 4 categories: none, ≤1 time per month, 2 to 4 times per month, and ≥2 times per week. Smoking status was divided into 3 groups: nonsmoker, past smoker, and current smoker. Past smokers who had quit smoking less than 1 year previously were included in the current smoker group. Participants were asked if they usually felt stressed and were divided into the following 4 groups based on reported stress levels: no stress, some stress, moderate stress, and severe stress. Participants under 110 cm or 30 kg were excluded from this study. Using the international classification of adults as underweight, overweight, and obese according to body mass index (BMI, kg/m^2^) defined by the World Health Organization,^18^ 4 BMI groups were devised: underweight, <18.5; healthy, ≥18.5, <25; overweight ≥25, <30; and obese, ≥30.

Participants were asked about their history regarding other comorbidities, such as hypertension, diabetes mellitus, hyperlipidemia, stroke, angina or myocardial infarction, arthritis, and asthma. Those who reported a history of any of these diseases, as diagnosed by a medical doctor, were recorded as positive for this category.

### Statistical Analysis

Differences in mean age, vigorous exercise days, and moderate exercise days between normal participants (control) and participants who fell were compared using linear regression analysis with complex sampling. The differences in rates of sex, hypertension, diabetes mellitus, hyperlipidemia, stroke, angina or myocardial infarction, arthritis, asthma history, income level, education level, smoking, alcohol consumption, hours of sleep, stress level, and BMI groups were compared using a Chi-square test with a Rao-Scott correction.

Associations between slips or falls (≥1 time per year) and sleep duration groups were analyzed using the following: simple logistic regression analysis with complex sampling (unadjusted); multiple logistic regression analysis with complex sampling adjusted by age and sex (model 1); and multiple logistic regression analysis with complex sampling adjusted by age, sex, vigorous exercise days, moderate exercise days, income level, education level, smoking, alcohol consumption, BMI group, stress level, hypertension, diabetes mellitus, hyperlipidemia, stroke, angina or myocardial infarction, arthritis, and asthma history (model 2). Secondary analysis was performed on participants with a history of slips or falls ≥2 times per year according to the same methods. In these analyses, the reference sleep duration was considered 7 hours per day because 7 hours of sleep was the largest sleep group recorded in this study (66,478 participants, 32.2%).

For subgroup analysis according to age, participants were divided into 3 groups based on age: 19 to 40 years old, 41 to 60 years old, and 61 + years old. A subgroup analysis of sleep duration and falls (≥1 time or ≥2 times) was performed using multiple logistic regression analysis with complex sampling (model 2).

To analyze the participants according to fall location, a subgroup analysis of sleep duration and falls (indoor or outdoor) was performed using multiple logistic regression analysis with complex sampling (model 2).

Two-tailed analyses were conducted, and *P*-values lower than 0.05 indicated significance. The adjusted odds ratio (AOR) and 95% confidence interval (CI) for fall down were calculated. All results are presented as weighted values. The results were analyzed statistically using SPSS ver. 21.0 (IBM, Armonk, NY).

## RESULTS

A total of 18.4% (168,362) of participants experienced slips or falls. There were various reasons of fall down (see Table S1, Supplemental Content, which illustrates reasons of fall down of participants). Specifically, 11.3% (23,316), 4.1% (8563), 1.7% (3502), 0.3% (650), and 1.0% (1989) of participants experienced falls 1, 2, 3, 4, and 5 or more than 5 times per year, respectively. Overall, the mean sleep duration was 6.60 ± 1.20 hours per day. A total of 16.4% (33,805), 29.8% (61,418), 32.2% (66,478), 18.2% (37,641), and 3.4% (7040) of participants reported sleep durations of 3 to 5, 6, 7, 8, and 9 to 12 hours per day, respectively. Participants with or without falls (≥1 time or ≥2 times per year) showed statistically significant differences by age, vigorous or moderate exercise days, sex, income, education, alcohol, stress levels, medical illness, and sleep duration (Table 1 ). These results warranted adjusting variables to analyze the relation between sleep duration and falls.

**TABLE 1:**
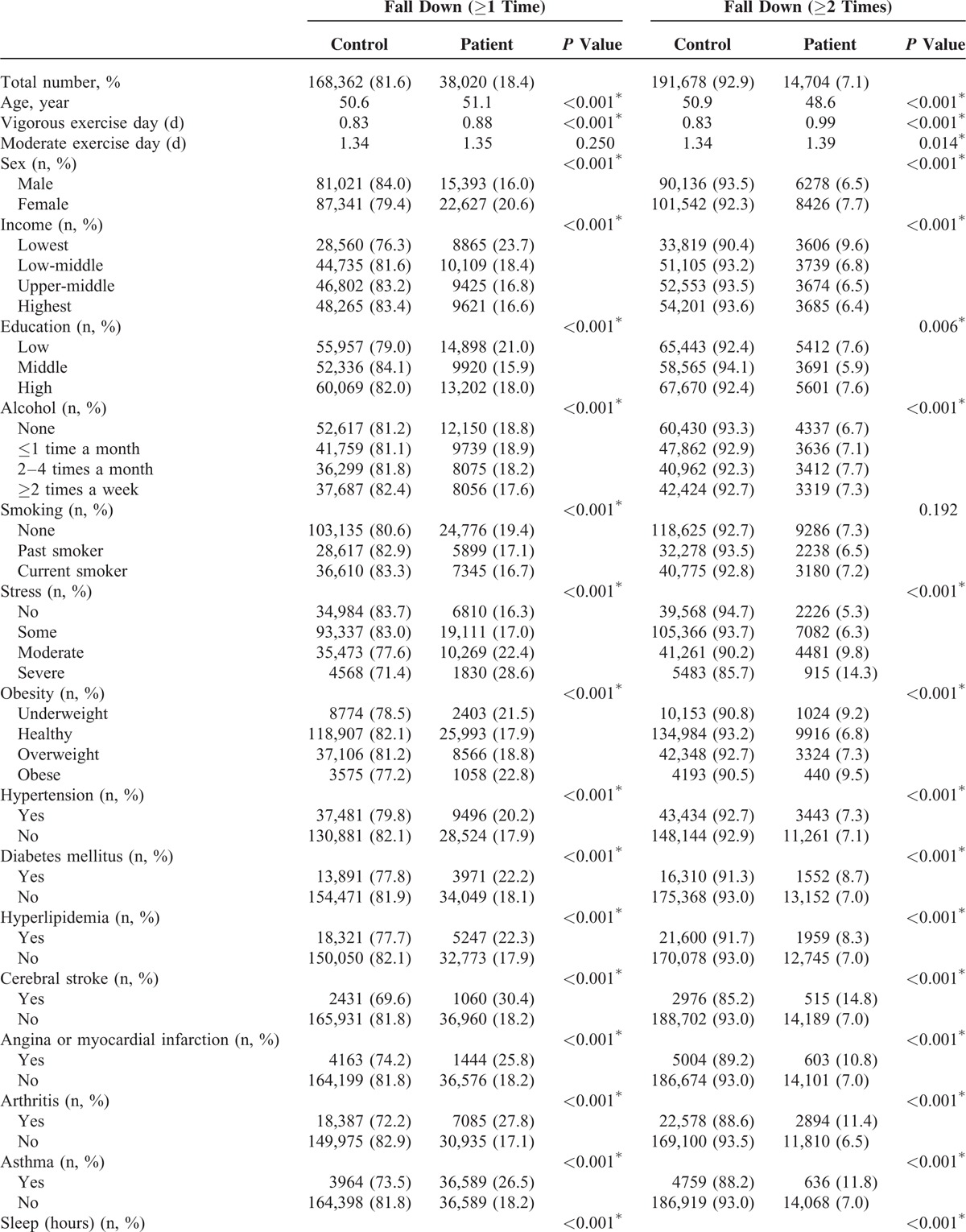
General Characteristic of Participants

**TABLE 1 (Continued):**
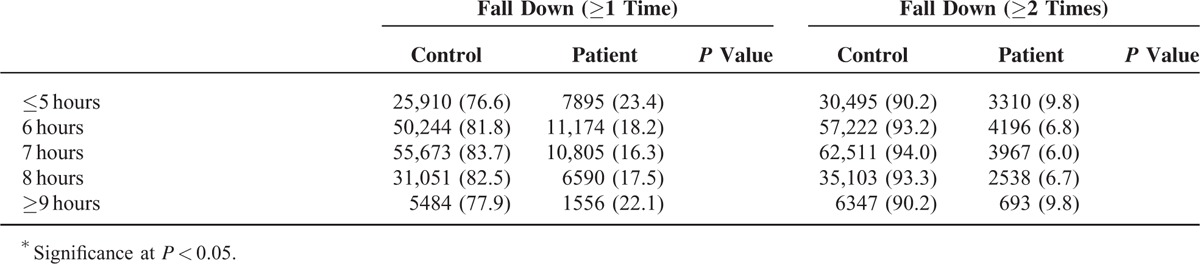
General Characteristic of Participants

Both ≤6 and ≥8 hours of sleep duration were significantly associated with experiencing falls (≥1 time and ≥2 times per year) in unadjusted, model 1 and model 2 (each at *P* < 0.001) (Table 2) analyses. Furthermore, the ORs and AORs for falls showed U-shaped correlations and a dose-response relationship with a sleep duration of 7 hours per day. In other words, the AOR for falls (≥2 times per year) and ≤5 hours of sleep duration (AOR = 1.47; 95% CI = 1.38–1.56; *P* = 0.007) was higher than that of 6 hours of sleep duration (AOR = 1.12; 95% CI = 1.06–1.18; *P* = 0.007). Similarly, the AOR for falls (≥2 times per year) and ≥9 hours of sleep duration (AOR = 1.38; 95% CI = 1.23–1.55; *P* = 0.007) was higher than that of 8 hours of sleep duration (AOR = 1.10; 95% CI = 1.03–1.17; *P* = 0.007). These U-shaped relations between sleep durations and falls were maintained even after excluding the participants who fall down by dizziness (see Table S2, Supplemental Content, which illustrates odds ratios of sleep time for fall down [≥1 time or ≥2 times] except for the participants who fall down by dizziness). Similarly, when the multiple logistic analyses were performed only in the participants without chronic diseases, the U-shaped relations between sleep durations and falls were still persistent (see Table S3, Supplemental Content, which illustrates odds ratios of sleep time for fall down [≥1 time or ≥2 times] in participants who have no chronic disease histories).

**TABLE 2:**
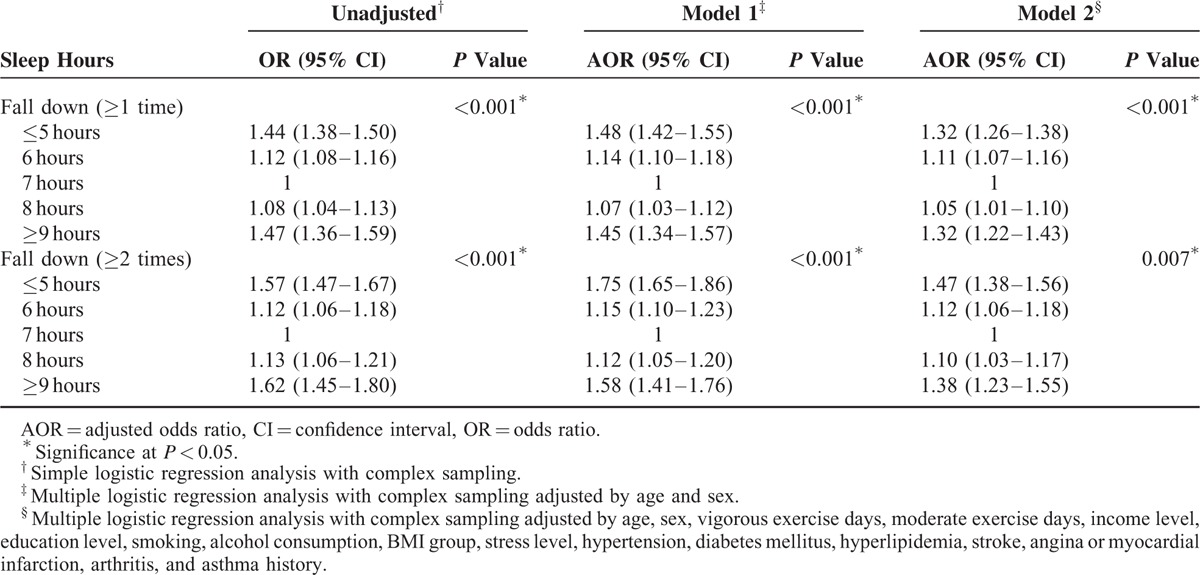
Odds Ratios of Sleep Time for Fall Down (≥1 Time or ≥2 Times) Using Simple and Multiple Logistic Regression Analyses With Complex sampling

In the subgroup analysis according to age groups, associations between sleep duration and incidences of falls showed similar patterns as those of the total study population (Table 3). Sleep durations of ≤5 or ≥9 hours per day were significantly associated with an increased incidence of falls (≥1 time or ≥2 times per year) in each age group. However, there were differences according to age group in the relation between falls and sleep duration. In the ≥61 year age group, 6 hours of sleep was not associated with falls (≥2 times per year). However, 6 hours of sleep was linked with falls (≥2 times per year) in both the 19 to 40 and 41 to 60 year age groups (19–40 years old, AOR of 6 hours of sleep = 1.18; 95% CI = 1.09–1.28; 41–60 years old, AOR of 6 hours of sleep = 1.12; 95% CI = 1.01–1.24). In the 19 to 40 year age group, a sleep duration of 9 hours per day was not significantly associated with the incidence of falls (≥2 times per year) in the 19 to 40 year age group but was significantly associated with falls (≥2 time a year) in both the 41 to 60 and ≥61 year age groups (41–60 years old, AOR of ≥9 hours of sleep = 1.32; 95% CI = 1.03–1.70; ≥61 years old, AOR of ≥9 hours of sleep = 1.43; 95% CI = 1.18–1.74). Sleep durations of 8 hours per day did not have a significant relation with the incidence of falls ≥2 times per year among all age groups.

**TABLE 3:**
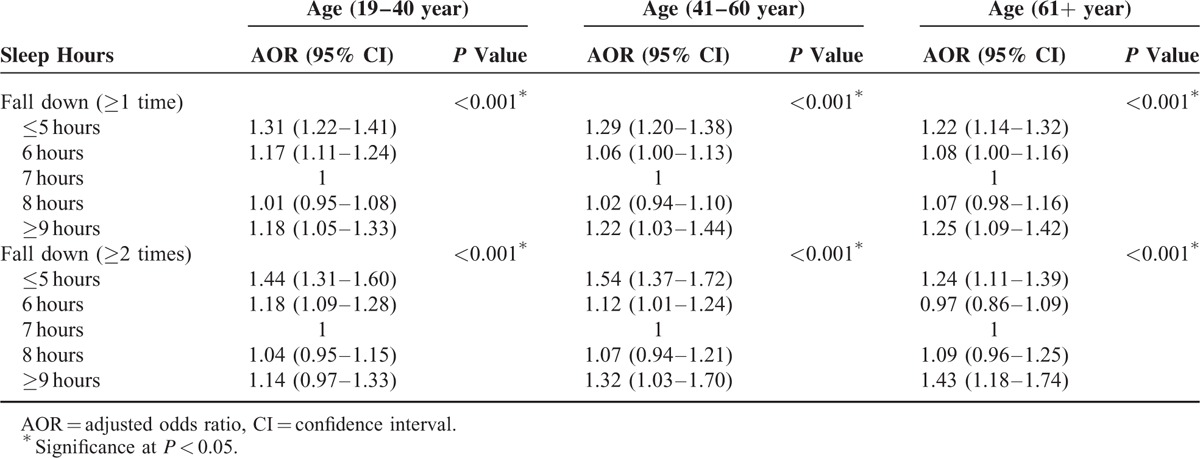
Subgroup Analysis of Sleep Time for Fall Down (≥1 Time or ≥2 Times) Using Multiple Logistic Regression Analysis With Complex Sampling (Model 2) According to Age Groups

The relations between falls (both indoor and outdoor) and sleep duration were analyzed according to age group. Both indoor and outdoor falls (≥1 time per year) were significantly associated with a sleep duration of ≤5 or ≥9 hours per day in all age groups (Table 4). Although both indoor and outdoor falls showed significant relations with sleep duration, outdoor falls were significantly associated with a sleep duration of 6 hours in the 41 to 60 year age group and with a sleep duration of 8 hours in the 19 to 40 and ≥61 year age groups. These same relations did not prove to be significant with regard to indoor falls (each *P* < 0.001). However, the AORs of indoor falls at ≥9 hours of sleep (AOR = 1.55; 95% CI = 1.27–1.90; *P* < 0.001) were higher than those of outdoor falls (AOR = 1.17; 95% CI = 1.01–1.36; *P* < 0.001).

**TABLE 4:**
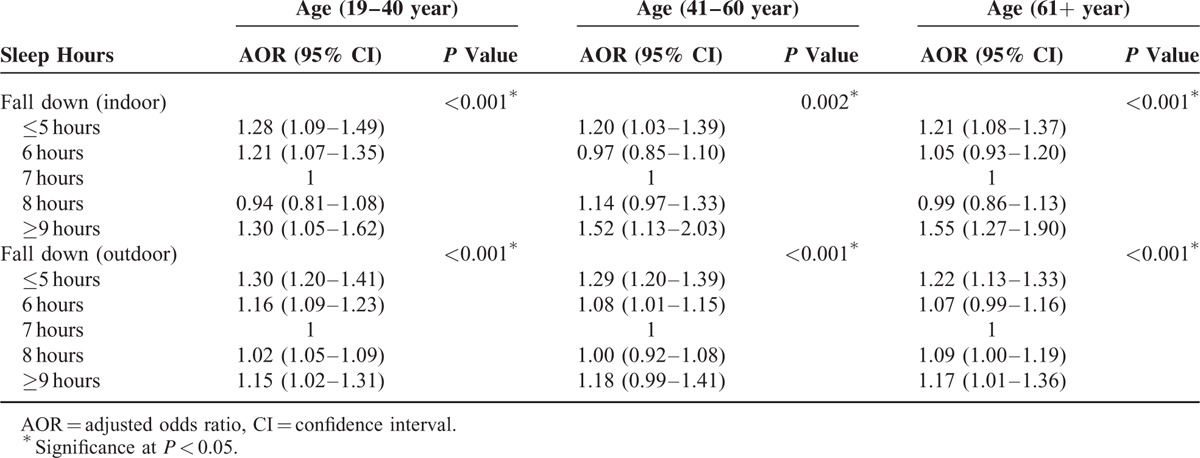
Subgroup Analysis of Sleep Time for Fall Down (Indoor or Outdoor) Using Multiple Logistic Regression Analysis With Complex Sampling (Model 2) According to Age Groups

## DISCUSSION

Falls are common, with an incidence of 18.4% in the present study. The incidence of falls was associated with sleep duration. The incidence of falls was lowest at a sleep duration of 7 hours per day and exhibited a dose-dependent, U-shaped correlation with the sleep duration with more than and less than 7 hours per day. Moreover, our data demonstrated age-specific differences influencing the impact of sleep duration on the incidence of falls. In the elderly, the short sleep durations resulted in a diminished association with falls compared to younger adults, who exhibited a significant association between sleep duration and falls (≥1 time or ≥2 times per year) when sleeping ≤5 per night. There were also differences in the associations between sleep duration and the incidence of outdoor versus indoor falls. These U-shaped correlations, age-specific relations, and differences in outdoor or indoor falls were consistent in our primary (falls of ≥1 time per year) and secondary (falls of ≥2 times per year) analyses. To our knowledge, research examining the relationship between these variables has not been conducted in previous studies.

Our previous work and other previous studies have reported that deprived sleep duration is associated with an increased incidence of falls.^7,19^ This increased incidence of falls resulting from short sleep duration may be explained by worsened attention, slower reaction times, and memory problems due to a sleep-deprived condition.^20^ In particular, the increased incidence of falls associated with sleep deprivation may have an increasingly detrimental effect on the elderly as sleep deprivation due to insomnia or sleep fragmentation increases with age. Additionally, other well-known risk factors that may increase risk of falls, such as osteoporosis, physical inactivity, and visual impairment, are more common among the elderly.^21,22^ Fortunately, our data demonstrated an attenuated relation between deprived sleep duration and the incidence of falls among the elderly.

Excessive sleep duration also caused a detrimental influence on the incidence of falls in this study. In accordance with our results, one recent study suggested that inadequate sleep times (both short and long) were significantly correlated with lower quality of life.^23^ One study also demonstrated that more than 9 hours of sleep per day was associated with an increased experience of falls in elderly.^24^ This detrimental effect resulting from excessive sleep duration on the incidence of falls can be explained by a lack of compensatory recruitment of brain activity following sleep deprivation in excessive sleepers. For instance, acute total sleep deprivation has been demonstrated to adaptively activate specific areas of the prefrontal cortex and parietal lobes, which are associated with lowered levels of memory impairment and increased responsiveness to certain cognitive tasks.^25,26^ Although there are conflicting reports in the literature, acute total sleep deprivation has also been reported to increase vestibulo-ocular responses.^27^ Therefore, we can presume that sufficient but not excessive sleep time is optimal for maintaining the adequate responsiveness and cognitive function needed to prevent falls.

This U-shaped correlation between sleep duration and the incidence of falls demonstrated some differences according to age. As noted above, short sleep duration was less significantly associated with falls in the elderly. Furthermore, falls among young adults were significantly correlated with slightly shorter sleep durations. Falls among young adults were not influenced by long sleep durations. These differences may be caused by the age-related changes in sleep patterns. As individuals age, sleep latency and the percentage of stage 1 and stage 2 sleep increase, whereas REM sleep decreases, which results in chronic shortness of sleep duration among the elderly as compared to younger adults.^28^ This pattern is in line with the differing recommendations for appropriate sleep durations according to age, with 7 to 9 hours recommended for young adult and middle-aged individuals, and 7 to 8 hours recommended for the elderly. Therefore, being elderly is a predictor of being more adapted to short sleep durations.

Our data also demonstrated that the effect of sleep duration on the incidence of falls was different in indoor versus outdoor falls. Both indoor and outdoor falls showed significant U-shaped relationships with sleep duration. It is possible that decreased reactiveness and attention due to inadequate sleep time may be more influential on outdoor falls, as there are many more stimuli present in outdoor situations as opposed to indoors.^20^ However, indoor falls showed higher AORs with sleep durations ≥9 hours, which implies that extremely long sleep duration has detrimental effects regarding both indoor and outdoor falls. This relation was likely caused by the fact that indoor circumstances are more susceptible to activity immediately after waking up, which begins after recovery from the diminished consciousness associated with sleep, as compared to outdoor circumstances.

Many studies have explored the effect of sleep parameters on the incidence of falls, but the results have varied. Sleep disturbances such as having a hard time falling or staying asleep were associated with falls in some studies but did not show a significant association in others.^29–31^ Plausible reasons for these discrepancies among previous studies include differences in study populations with sleep disturbances and other sleep-related comorbidities and differences in the sleep-related parameters collected and the covariates involved in the analysis. In this respect, there are certain limitations in the present study, which originated from a cross-sectional, survey-based study. First, although we adjusted for numerous covariates, including age and sex, the degree of physical activity, income, education level, alcohol use, smoking, stress, and medical illness, several possible confounding factors were not considered in this study. For example, detrimental health behaviors and particular lifestyles, such as unmarried status, irregular dietary habits, depression, history of dizziness or balance disorder, and medication history may be correlated with abnormal sleep duration.^32,33^ However, we demonstrated that the U-shaped relationships between sleep durations and falls were consistent after excluding the participants who fall down by dizziness (see Table S2, Supplemental Content, which illustrates odds ratios of sleep time for fall down (≥1 time or ≥2 times) except for the participants who fall down by dizziness) or had a history of chronic diseases who might have the higher chance of taking medications (see Table S3, Supplemental Content, which illustrates odds ratios of sleep time for fall down (≥1 time or ≥2 times) in participants who have no chronic disease histories). Therefore, these confounding factors might have little effect to the results of our study. In addition, we could not assess various sleep-related parameters, such as sleep structure, fluctuation of sleep time, sleep quality, insomnia, sleep apnea, and sleep medication. There was also a potential recall bias with sleep duration, as sleep duration was subjectively collected based on the responses of participants. Moreover, the sleep duration was investigated in 1 hour intervals, which may limit the accuracy of the recorded sleep duration. Finally, although this study was based on a large representative population of Koreans, sample sizes were reduced for subgroup analysis, which may influence the statistical significance of the subgroup analysis. For example, both indoor and outdoor falls according to age group might be small sample sizes for demonstrating statistical power, even though there were clinically significant relations with sleep duration. To further elucidate the causal relation between sleep duration and falls, additional studies based on more numerous sleep parameters and implemented using prospective study designs are warranted.

This study demonstrated a U-shaped relation between sleep duration and the incidence of falls in Korean adults. The group with 7 hours of sleep duration showed the lowest incidence of falls. This U-shaped relation manifested in some age-specific characteristics. The elderly showed an attenuated relation between falls and short sleep duration, whereas young adults showed an attenuated relation between falls and long sleep duration. Because sleep duration is a modifiable factor, information on the effect of sleep duration on the incidence of falls and subsequent efforts to maintain adequate sleep duration will have great clinical implications for the prevention or reduction of the incidence of falls.

## Supplementary Material

Supplemental Digital Content
