# Supplementary material for: Excessive Sleep and Lack of Sleep Are Associated With Slips and Falls in the Adult Korean Population: A Population-Based Cross-Sectional Study
Source: Medicine (Baltimore). 2016 Jan 29;95(4):e2397. doi: 10.1097/MD.0000000000002397 (PMC5291551; doi:10.1097/MD.0000000000002397)
Supplement: Supplemental Digital Content [file medi-95-e2397-s001.doc]

Supplement Table S1. Reasons of fall down of participants.

| Reason of fall down | Fall down ≥ 1 time | | Fall down ≥ 2 times | |
| --- | --- | --- | --- | --- |
|  | N | % | N | % |
| Slippery floor | 21389 | 33.7 | 6788 | 26.7 |
| Bump into man or objects | 6435 | 10.1 | 2863 | 11.2 |
| Fall by door sill | 10449 | 16.5 | 4562 | 17.9 |
| Steep slope | 2285 | 3.6 | 1063 | 4.2 |
| Low light | 768 | 1.2 | 363 | 1.4 |
| Miss one’s step | 14128 | 22.3 | 6182 | 24.3 |
| Feel dizziness | 4978 | 7.8 | 2448 | 9.6 |
| Others | 3044 | 4.8 | 1193 | 4.7 |

Supplement Table S2. Odds ratios of sleep time for fall down (≥ 1 time or ≥ 2 times) except for the participants who fall down by dizziness using multiple logistic regression analyses (model 2) with complex sampling

| Sleep Hours | | AOR (95% CI) | P Value |
| --- | --- | --- | --- |
| **Fall Down (≥ 1 time)** | |  | <0.001* |
|  | ≤5h | 1.31(1.25-1.37) |  |
|  | 6h | 1.12(1.07-1.16) |  |
|  | 7h | 1 |  |
|  | 8h | 1.04(1.00-1.09) |  |
|  | ≥9h | 1.29(1.19-1.40) |  |
| **Fall Down (≥ 2 times)** | |  | <0.001* |
|  | ≤5h | 1.47(1.37-1.56) |  |
|  | 6h | 1.13(1.07-1.20) |  |
|  | 7h | 1 |  |
|  | 8h | 1.09(1.02-1.17) |  |
|  | ≥9h | 1.35(1.20-1.51) |  |

*Significance at P < 0.05.

Supplement Table S3. Odds ratios of sleep time for fall down (≥ 1 time or ≥ 2 times) in participants who have no chronic disease histories using multiple logistic regression analyses (model 2) with complex sampling

| Sleep Hours | | AOR (95% CI) | P Value |
| --- | --- | --- | --- |
| **Fall Down (≥ 1 time)** | |  | <0.001* |
|  | ≤5h | 1.32(1.25-1.39) |  |
|  | 6h | 1.14(1.09-1.19) |  |
|  | 7h | 1 |  |
|  | 8h | 1.05(1.00-1.11) |  |
|  | ≥9h | 1.29(1.17-1.43) |  |
| **Fall Down (≥ 2 times)** | |  | <0.001* |
|  | ≤5h | 1.49(1.37-1.62) |  |
|  | 6h | 1.17(1.10-1.26) |  |
|  | 7h | 1 |  |
|  | 8h | 1.09(1.00-1.17) |  |
|  | ≥9h | 1.32(1.14-1.52) |  |

*Significance at P < 0.05.
